# Supplementary material for: Identifying and analyzing sepsis states: A retrospective study on patients with sepsis in ICUs
Source: PLOS Digit Health. 2022 Nov 10;1(11):e0000130. doi: 10.1371/journal.pdig.0000130 (PMC9931346; doi:10.1371/journal.pdig.0000130)
Supplement: S1 Text — (PDF) [file pdig.0000130.s001.pdf]

Supplementary Material

Analyses of other variables.

Arterial blood gas (ABG). ABG is a blood test that assesses the gas exchange and acid-base balance of the body. It is an essential marker for critical patients admitted to ICUs. Arterial PH, PaO2, PaCO2, HCO3, and Arterial base excess are the main components of ABG. The presence of acidosis (arterial PH less than 7.35) or alkalosis (arterial PH higher than 7.45) is assessed by measuring arterial PH in the blood. Combined with arterial pH and PaCO2, one can measure the existence of respiratory acidosis or respiratory alkalosis in the body. Respiratory acidosis occurs when PaCO2 is higher than 45 mmHg, with an arterial PH less than 7.35. Respiratory alkalosis occurs when PaCO2 is less than 35 mmHg, with an arterial PH higher than 7.45. Combined with arterial PH and HCO3, one can measure the existence of metabolic acidosis or metabolic alkalosis. Metabolic acidosis occurs when HCO3 is less than 22 mEq/L, with an arterial PH less than 7.35. Metabolic alkalosis occurs when HCO3 is higher than 28 mmHg, with an arterial PH higher than 7.45. Among four types of acid-base disorders (respiratory acidosis, respiratory alkalosis, metabolic acidosis, and metabolic alkalosis), metabolic acidosis is common in sepsis patients with organ failure [1]. Metabolic alkalosis has been noted in sepsis patients [2]. In our cohort, we find that the average values of arterial PH and HCO3 in MODS states are lower than non-MODS states, indicating that a higher portion of patients with metabolic acidosis is observed in MODS states. The average values of arterial PH for states A1 through A6 are 7.39, 7.40, 7.39, 7.35, 7.37, and 7.36, respectively. The average values of HCO3 for states A1 through A6 are 24.68, 25.06, 24.47, 22.79, 23.08, and 21.93, respectively. The percentage of cases with metabolic acidosis in states A1 through A6 are 8.7%, 6.6%, 6.7%, 22.8%, 16.4%, and 20.5%, respectively. On the other hand, we observe fewer cases of metabolic alkalosis in our cohort. The percentage of cases with metabolic alkalosis in states A1 through A6 are 4.5%, 6.2%, 3.9%, 4.7%, 2.6%, and 1.1%, respectively.

Arterial base excess (Arterial BE). Arterial BE reflects the metabolic component of the acid-base balance. Arterial BE measures the amount of H+ required to return the blood PH to normal when PaCO2 is within the normal range, with the normal

range from -2 to +2. The base excess increases in metabolic alkalosis and decreases (or becomes negative) in metabolic acidosis. Metabolic acidosis is common in sepsis patients with organ failure [1], and metabolic alkalosis can also occur in sepsis patients [2]. In our study, we find that the average value of arterial BE in MODS states is negative, indicating an inclination towards metabolic acidosis in MODS states. The average values of arterial BE for states A1 through A6 are 0.36, 0.80, 0.37, -1.92, -1.94, and -2.46, respectively, and the percentage of cases with metabolic acidosis in states A1 through A6 are 8.7%, 6.6%, 6.7%, 22.8%, 16.4%, and 20.5%, respectively. Therefore, arterial BE is an important predictor of metabolic acidosis for sepsis patients.

Albumin. Albumin is one of the essential proteins, with a normal range of 3.4 to 5.4 g/dl. Albumin is responsible for plasma colloid osmotic pressure, acting as a major binding protein for endogenous and exogenous compounds (drugs), with antioxidant and anti-inflammatory properties, and operates as a buffer to balance acid-base status of the body. A lower albumin level (Hypoalbuminemia) is often observed when: (i) patients have nutritional deficiencies [3]; (ii) patients develop chronic liver disease, advanced hepatic cirrhosis, or end-stage renal disease [4]; or (iii) an inflammation is present [5]. Albumin, in addition to crystalloids, is often used for initial resuscitation and subsequent intravascular volume replacement in patients with sepsis and septic shock [6]. Although albumin administration is widely used in the management of sepsis, the benefit of the use of albumin for resuscitation in this population remains controversial – while several meta-analyses have shown that the administration of albumin in ICU patients has beneficial effects on health outcomes [7, 8], other studies have shown contradictory results [9]. However, the results are less conclusive when the included studies have differing experimental design and comparison groups. As a result, albumin administration is suggested with weak confidence [6]. In our study, we find hypoalbuminemia in all sepsis states, and the albumin level in various sepsis states is not significantly different. Future development of clinical trials may focus on comparing the effects of albumin administration on the health outcomes for different sepsis states.

Hemoglobin (Hb). The normal range of hemoglobin for males and females is 13.5 to 17.5 grams per deciliter and 12.0 to 15.5 grams per deciliter, respectively. A lower

hemoglobin level in the body indicates a low red blood cell (RBC) count (Anemia). Anemia is common in sepsis due to inflammation, liver and renal impairment, and cancer. RBC transfusion is strongly recommended for patients with sepsis when hemoglobin concentration falls to less than 7.0 g/dL in adults in the absence of extenuating circumstances, such as myocardial ischemia, severe hypoxemia, or acute hemorrhage [6]. We find that the average Hb values in all sepsis states are lower than the normal range, with A2 (Inflammation state) having the lowest Hb values. The average Hb values for states A1 through A6 are 10.30, 9.49, 10.49, 10.42, 10.90, and 10.75, respectively.

Shock index (SI). Shock index (SI) is a bedside assessment defined as heart rate (HR) divided by systolic blood pressure (SysBP), with a normal range of 0.5 to 0.7 in healthy adults. SI is suggested as a measure in the triage and management of critically ill patients. Its use is also suggested as a predictor of clinical outcomes, such as the serum lactate level in the body, the risk of mortality, and other markers of morbidity [10]. However, a retrospective database review shows that SI does not correlate with the mortality rate in emergency room patients [11]. Our results support this claim. We find that SI is not highly associated with worse outcomes. While the MODS states display higher SI than A1 and A3 states, A2 (Inflammation state) displays the highest SI, which is the state associated with lower SOFA score and mortality rate. The average SI values for states A1 through A6 are 0.75, 0.81, 0.72, 0.77, 0.79, and 0.77, respectively. Furthermore, SI is not an independent predictor of hyperlactatemia (serum lactate $\geq$ 4.0 mmol/L): while A2 state manifests the highest average SI, it displays the lowest arterial lactate. The average arterial lactate levels for states A1 through A6 are 2.04, 1.88, 2.10, 5.50, 3.95, and 4.58.

Ionized calcium. In health, serum ionized calcium concentration is maintained between approximately 1.16 and 1.32 mmol/L. Ionized hypocalcemia (ionized calcium levels $<$ 1.16 mmol/L) are common in critically ill patients with sepsis, cardiac failure, pulmonary failure renal failure, post-surgery or burns [12]. Recent studies show that low ionized calcium concentrations coincide with increased severity of illness and increased mortality [12]. We find that ionized hypocalcemia occurs in all sepsis states, and the

average ionized calcium concentration in MODS states is lower than non-MODS states. The average ionized calcium concentration for states A1 through A6 are 1.13, 1.13, 1.14, 1.08, 1.07, and 1.09, respectively.

Calcium. The normal range of total serum calcium concentration is 8.8 mg/dL to 10.7 mg/dL. Hypocalcemia, defined as serum calcium concentration less than 8.8 mg/dL or serum ionized calcium concentration less than 4.7 mg/dL, is common in critically ill patients, especially in those with sepsis [12]. We find that hypocalcemia occurs in all sepsis states. However, low serum calcium concentrations do not coincide with the increased severity of illness and increased mortality. The serum calcium concentrations for states A1 through A6 are 8.31, 8.39, 8.38, 8.50, 8.13, and 8.20, respectively.

Magnesium. Magnesium is a vital element involved in various physiological processes and an essential cofactor in more than 300 enzymes, with a normal range of 1.5 to 2.5 mEq/L in healthy adults. Hypomagnesemia (serum Mg levels $<$ 1.5 mEq/L) can occur in critically ill patients, including sepsis patients, and is associated with prolonged ICU stay, increased need for mechanical ventilation, and increased mortality. In our study, we find that, in the average case, serum magnesium levels in all sepsis states are within the normal range. The average values of serum magnesium levels for states A1 through A6 are 2.06, 2.07, 2.02, 2.10, 2.04, and 2.29, respectively. We note that recent studies have shown that the administration of magnesium sulfate increases lactate clearance in critically ill patients with severe sepsis [13], improve cerebral perfusion in patients with sepsis-associated encephalopathy (SAE) [14], and may be used to open up small vessels, to reduce organ failure for patients with severe sepsis and septic shock [15]. Future development of clinical trials may focus on comparing the effects of the administration of magnesium sulfate on the health outcomes for different sepsis states.

Chloride. Chloride is an essential anion of the extracellular fluid, representing two-thirds of all negative charges in plasma and accounting for nearly one-third of plasma tonicity. Normal Serum chloride concentrations range from 96 to 106. mEq/L. Abnormal chloride levels in the blood (hypo- and hyperchloremia) are observed in critically ill patients. However, evidence on the effects of hypo- and hyperchloremia on the clinical outcomes, such as length of stay and mortality rate, are sparse [16]. A

recent study shows that hyperchloremia is not significantly related to an increased mortality rate, and hypochloremia is associated with increased mortality in patients with severe sepsis or septic shock [17]. Our results are consistent with these findings: we observe a higher percentage of hypochloremia in MODS groups, which are the groups associated with a higher mortality rate. We also observe that hyperchloremia does not directly correlate with MODS groups. The percentage of cases with hypochloremia for states A1 through A6 are 7%, 7%, 5%, 15%, 12%, and 10%, respectively, and the percentage of cases hyperchloremia for states A1 through A6 are 39%, 27%, 36%, 30%, 35%, and 35%, respectively.

Sodium. The normal sodium level in the blood is 135 to 145 mEq/L. Hypernatremia (serum sodium concentration $>$ 145 mEq/L) is an uncommon but important electrolyte abnormality in ICU patients. Hypernatremia also occurs in sepsis patients, but only a few studies [18, 19] have investigated the effect of serum sodium levels on the clinical outcomes in sepsis patients. Studies have shown that patients admitted with hypernatremia are significantly more likely to have sepsis [18] and that hypernatremia is strongly associated with worse outcomes in sepsis [19]. However, we notice that sepsis patients with hypernatremia only constitutes 7.3% in the cohort and that although we find that state A4 (the state with highest mortality rate) constitutes the highest portion of patients with hypernatremia, state A5 (the state with second-highest mortality rate) has a lower portion of patients with hypernatremia than the cohort.

The percentage of cases with hypernatremia for states A1 through A6 are 7.3%, 8.1%, 4.1%, 10.8%, 4.5%, and 8.8%, respectively.

Potassium. Potassium is one of the electrolytes mostly present in intracellular fluid. The normal value of serum potassium is 3.5 to 5.0 mEq/L. Potassium homeostasis is important for negative resting membrane potential, neuromuscular, and cardiac excitability. Abnormal potassium has adverse effects on the heart: both hypo and hyperkalemia cause cardiac arrhythmia. Hypokalaemia also causes muscle paralysis, including respiratory muscles and GIT. Potassium abnormality can occur in critically ill patients in ICU due to organ derangement and some medications, and is associated with an increased complication rate and mortality risk . Our study found that the average

potassium levels do not vary across sepsis states and are within the normal range in our cohort. The average potassium concentration for states A1 through A6 are 4.08, 4.22, 4.14, 4.31, 4.06, and 4.34, respectively.

SOFA and SIRS scores.

Systemic Inflammatory Response Syndrome (SIRS). *Sepsis* was first defined as a systemic inflammatory response syndrome (SIRS) [20]. SIRS is the clinical presentation of the host response to inflammation. It manifests in four symptoms, temperature $\geq$ 38 degree Celsius or $\leq$ 36 degree Celsius, respiratory rate $\geq$ 20 breaths/minute or $PaCO_2 < 32$ mm of Hg, heart rate $>$ 90 beats/minute, white blood count >12000/mm3 or $< 4000/mm^3$ or bands $>$ 10%. Two or more symptoms of SIRS indicate a SIRS positive case. However, it was argued that SIRS is not an adequate measure, since a sepsis-related symptom may be observed without infection. Due to its non-specific issue, the diagnostic metric of sepsis was first replaced by sepsis-2 [21], and finally changed to Sequential Organ Failure Assessment Score (SOFA).

Sequential Organ Failure Assessment [22] (SOFA) Score. SOFA measures the functionality of six organ systems – respiratory, coagulation, cardiovascular, neurological, liver, and renal, shown in S1 Table, each of which is measured by PaO_2/FiO_2 ratio, platelet count, mean arterial pressure (MAP), Glasgow coma score (GCS), bilirubin, and creatinine or urine output, respectively. Each system is assigned a score from 0 to 4. The worst condition represents the highest score. The range of the SOFA score is 0-24. It has been shown that the SOFA score is a good predictor of mortality in intensive care units [23, 24].

Performance analysis for the feature selection methods.

We compare the top 15 features selected by (i)$Q_j(P_k)$ method measuring the ratio of inter-cluster inertia at the i-th feature B_i to the total inertia at the i-th feature T_i, (ii) $Q'_j(P_k)$ method measuring the ratio of the between-cluster inertia at i-th feature B_i to the total between-cluster inertia $\sum_{i=1}^{p} B_i$, (iii) and the feature that has a lower probability of overlapping between clusters.

The selected features are shown in S5 Table . We observe that the first five features selected by $Q_j(P_k)$ and $Q'_j(P_k)$ are identical – SGOT, SGPT, PaO2/FiO2, PaO2, and Platelet Count, in the same order, except for PaO2 and Platelets. For the rest of the features selected by $Q_j(P_k)$ and $Q'_j(P_k)$, PT, WBC Count, Arterial Lactate, Age, and HR were selected by both $Q_j(P_k)$ and $Q'_j(P_k)$. Among the 10 features mentioned above, 8 are selected by the variation test as well. We present a Venn diagram for the inclusion-exclusion comparison between $Q_j(P_k)$, $Q'_j(P_k)$, and the variation test in Fig 3 . We observe that the three methods are largely consistent in selecting representative features from the clusters.

Consistency analysis for archetypal analysis.

We regarded each time point as a snapshot of patient status and used archetypal analysis to find distinct states, and higher-order transition probabilities across states identified through archetypal analysis. The validity of transitions primarily relies on the stability or consistency of finding sepsis states from archetypal analysis. If the identification of sepsis state for each time point is significantly different for different runs, the validity of sepsis state identification and that of the constructed temporal models suffers. To test the stability of archetypal analysis, we used archetypal analysis on the sepsis dataset 20 times to compare the consistency of results between runs. Archetypal analysis is an unsupervised technique. Thus, we chose Normalized Mutual Information (NMI) [25] and Adjusted Rand Index (ARI) [26] as the consistency metric and measured how well the identified state for each time point has a one-to-one mapping relationship across runs. The average value of NMI and ARI are 0.9961 and 0.9732 with standard deviations 0.0011 and 0.0052, respectively.

Supplementary References

1. Kellum J, et al. Metabolic acidosis in patients with sepsis: epiphenomenon or part of the pathophysiology? Critical Care and Resuscitation. 2004;6(3):197.

2. Kreü S, Jazrawi A, Miller J, Baigi A, Chew M. Alkalosis in critically ill patients with severe sepsis and septic shock. PloS one. 2017;12(1):e0168563.

3. Keller U. Nutritional laboratory markers in malnutrition. Journal of clinical medicine. 2019;8(6):775.

4. Gatta A, Verardo A, Bolognesi M. Hypoalbuminemia. Internal and emergency medicine. 2012;7(3):193–199.

5. Don BR, Kaysen G. Poor nutritional status and inflammation: serum albumin: relationship to inflammation and nutrition. In: Seminars in dialysis. vol. 17. Wiley Online Library; 2004. p. 432–437.

6. Rhodes A, Evans LE, Alhazzani W, Levy MM, Antonelli M, Ferrer R, et al. Surviving sepsis campaign: international guidelines for management of sepsis and septic shock: 2016. Intensive care medicine. 2017;43(3):304–377.

7. Vincent JL, De Backer D, Wiedermann CJ. Fluid management in sepsis: the potential beneficial effects of albumin. Journal of critical care. 2016;35:161–167.

8. Xu JY, Chen QH, Xie JF, Pan C, Liu SQ, Huang LW, et al. Comparison of the effects of albumin and crystalloid on mortality in adult patients with severe sepsis and septic shock: a meta-analysis of randomized clinical trials. Critical Care. 2014;18(6):702.

9. Patel A, Laffan MA, Waheed U, Brett SJ. Randomised trials of human albumin for adults with sepsis: systematic review and meta-analysis with trial sequential analysis of all-cause mortality. Bmj. 2014;349:g4561.

10. Tseng J, Nugent K. Utility of the shock index in patients with sepsis. The American Journal of the Medical Sciences. 2015;349(6):531–535.

11. Liu Yc, Liu Jh, Fang ZA, Shan Gl, Xu J, Qi Zw, et al. Modified shock index and mortality rate of emergency patients. World journal of emergency medicine. 2012;3(2):114.

12. Müller B, Becker K, Kränzlin M, Schächinger H, Huber P, Nylen E, et al. Disordered calcium homeostasis of sepsis: association with calcitonin precursors. European journal of clinical investigation. 2000;30(9):823–831.

13. Noormandi A, Khalili H, Mohammadi M, Abdollahi A. Effect of magnesium supplementation on lactate clearance in critically ill patients with severe sepsis: a randomized clinical trial. European Journal of Clinical Pharmacology. 2020;76(2):175–184.

14. Shaban N, Helmy T, Al Awady S, Zidan D, et al. The effect of magnesium sulfate on cerebral perfusion in patients with sepsis-associated encephalopathy. Research and Opinion in Anesthesia and Intensive Care. 2019;6(3):300.

15. Pranskunas A, Vellinga NA, Pilvinis V, Koopmans M, Boerma EC. Microcirculatory changes during open label magnesium sulphate infusion in patients with severe sepsis and septic shock. BMC anesthesiology. 2011;11(1):12.

16. Filis C, Vasileiadis I, Koutsoukou A. Hyperchloraemia in sepsis. Annals of intensive care. 2018;8(1):43.

17. Oh HJ, Kim SJ, Kim YC, Kim EJ, Jung IY, Oh DH, et al. An increased chloride level in hypochloremia is associated with decreased mortality in patients with severe sepsis or septic shock. Scientific reports. 2017;7(1):1–9.

18. De Freitas G, Gudur A, Vela-Ortiz M, Jodelka J, Livert D, Krishnamurthy M. Where there is sodium there may be sepsis. Journal of community hospital internal medicine perspectives. 2019;9(4):296–299.

19. Ni Hb, Hu Xx, Huang Xf, Liu Kq, Yu Cb, Wang Xm, et al. Risk factors and outcomes in patients with hypernatremia and sepsis. The American Journal of the Medical Sciences. 2016;351(6):601–605.

20. Bone RC, Balk RA, Cerra FB, Dellinger RP, Fein AM, Knaus WA, et al. Definitions for sepsis and organ failure and guidelines for the use of innovative therapies in sepsis. Chest. 1992;101(6):1644–1655.

21. Levy MM, Fink MP, Marshall JC, Abraham E, Angus D, Cook D, et al. 2001 sccm/esicm/accp/ats/sis international sepsis definitions conference. Intensive care medicine. 2003;29(4):530–538.

22. Singer M, Deutschman CS, Seymour CW, Shankar-Hari M, Annane D, Bauer M, et al. The third international consensus definitions for sepsis and septic shock (Sepsis-3). Jama. 2016;315(8):801–810.

23. Jentzer JC, Bennett C, Wiley BM, Murphree DH, Keegan MT, Gajic O, et al. Predictive value of the sequential organ failure assessment score for mortality in a contemporary cardiac intensive care unit population. Journal of the American Heart Association. 2018;7(6):e008169.

24. Jones AE, Trzeciak S, Kline JA. The Sequential Organ Failure Assessment score for predicting outcome in patients with severe sepsis and evidence of hypoperfusion at the time of emergency department presentation. Critical care medicine. 2009;37(5):1649.

25. Estévez PA, Tesmer M, Perez CA, Zurada JM. Normalized mutual information feature selection. IEEE Transactions on neural networks. 2009;20(2):189–201.

26. Milligan GW, Cooper MC. A study of the comparability of external criteria for hierarchical cluster analysis. Multivariate behavioral research. 1986;21(4):441–458.
