# Supplementary figures and images for: Identifying and analyzing sepsis states: A retrospective study on patients with sepsis in ICUs
Source: PLOS Digit Health. 2022 Nov 10;1(11):e0000130. doi: 10.1371/journal.pdig.0000130 (PMC9931346; doi:10.1371/journal.pdig.0000130)

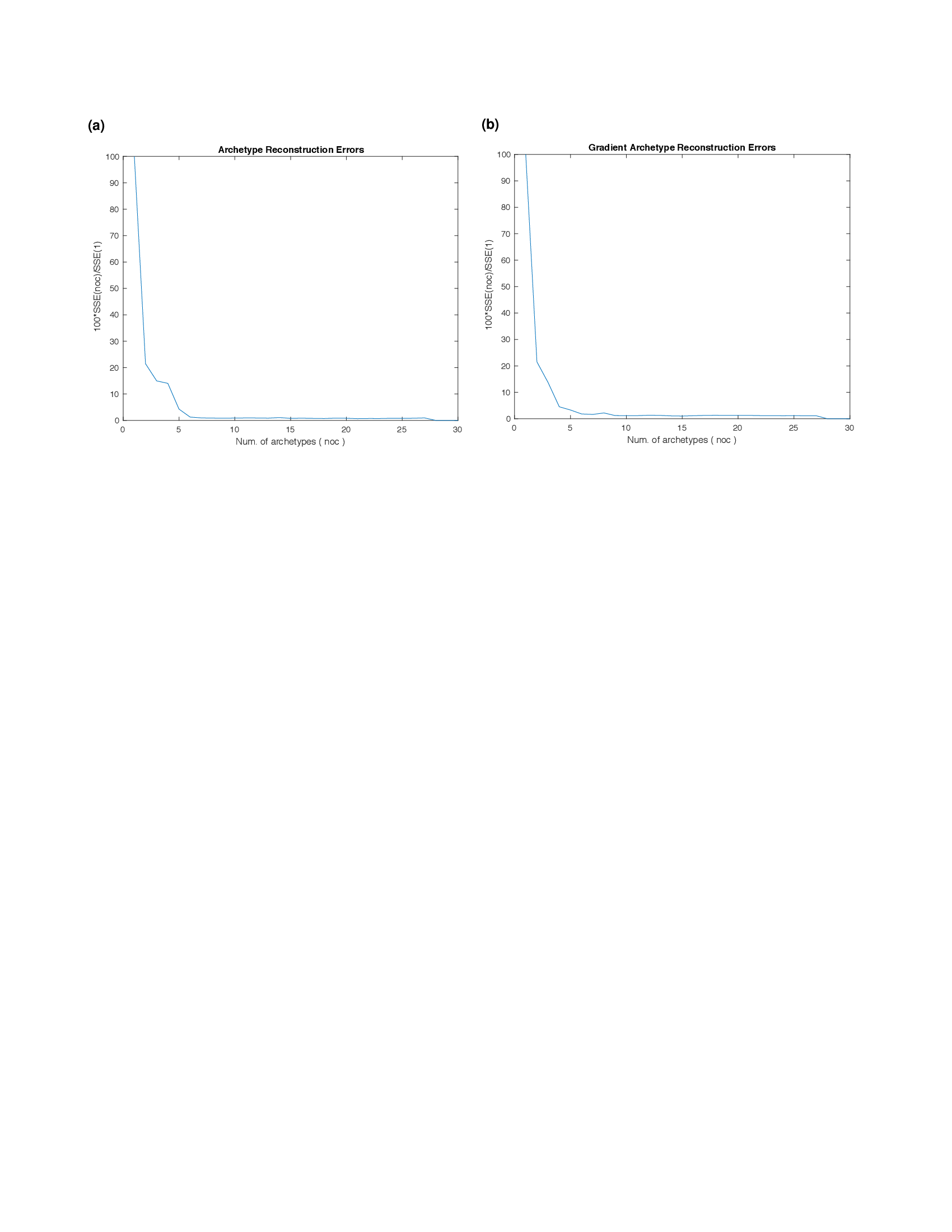

Supplement: S1 Fig — (A) Elbow method for finding optimal number of archetypes. (B) Elbow method for finding optimal number of gradient archetypes. (TIFF) [file pdig.0000130.s002.tiff]

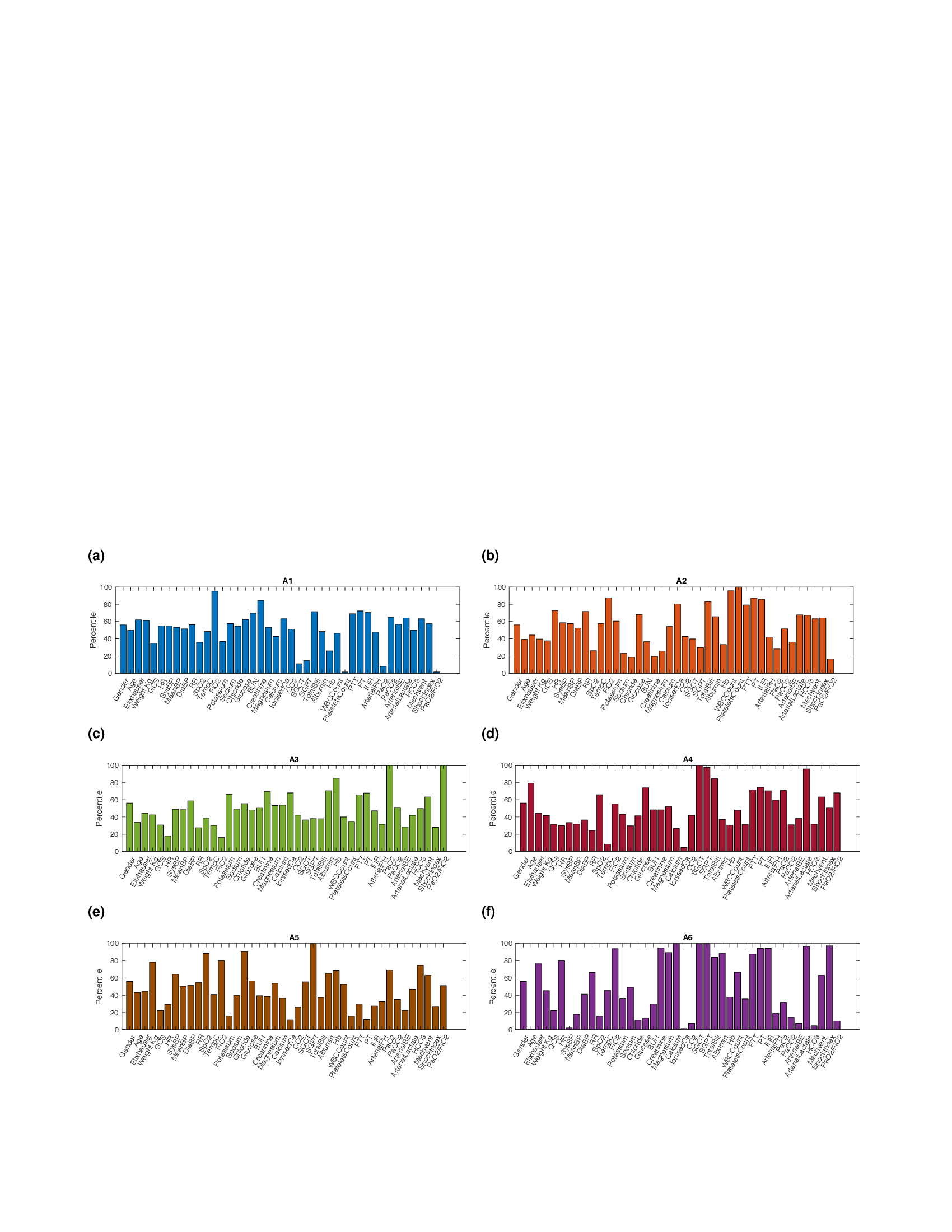

Supplement: S2 Fig — (TIFF) [file pdig.0000130.s003.tiff]

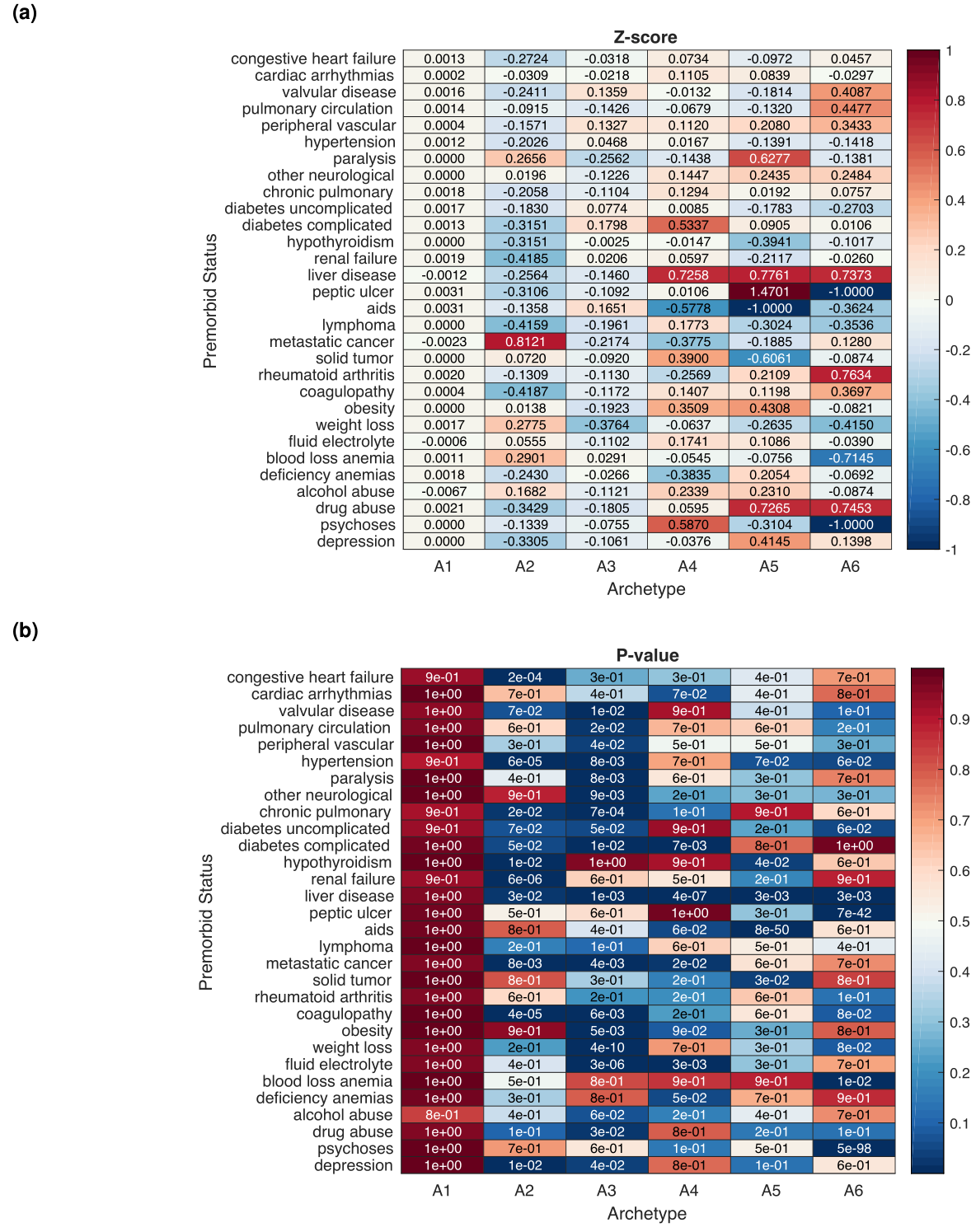

Supplement: S3 Fig — (A) Z-score analysis of comorbidity profiles (row) of each sepsis type (column). Entries approaching red in intensity indicate that the comorbidity profiles are expressed in the corresponding sepsis states, and entries closer to blue indicate that the comorbidity profiles are suppressed in corresponding sepsis states. (B) P-values for the pairwise two-sample t-test for the comorbidity profiles (row) of each sepsis type (column). Statistically Significant entries approach blue. (TIFF) [file pdig.0000130.s004.tiff]

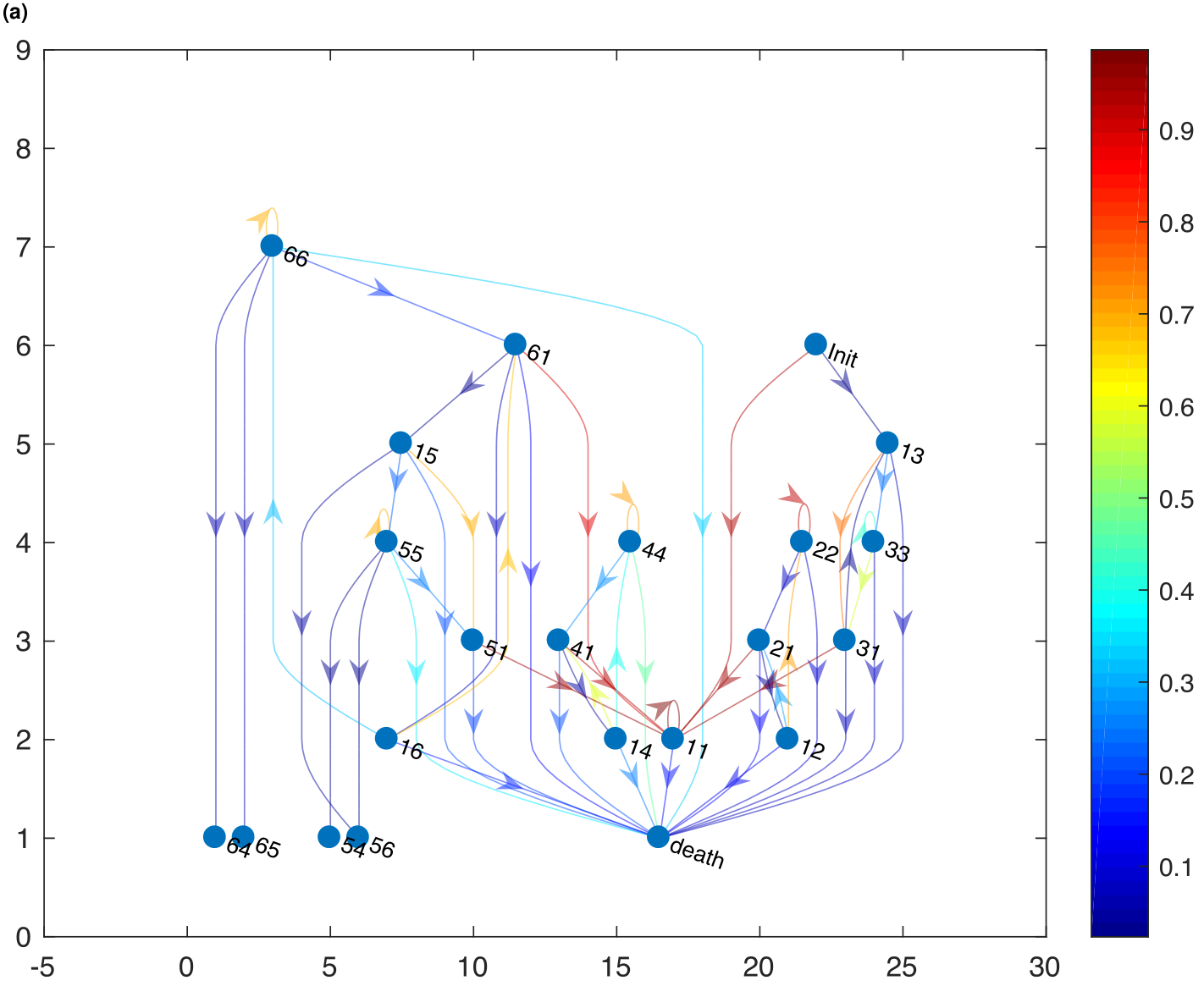

Supplement: S4 Fig — (A) Second-order transition graph: Edges approaching red in color indicate higher transition probabilities, and edges approaching black indicate lower transition probabilities. (TIFF) [file pdig.0000130.s005.tiff]

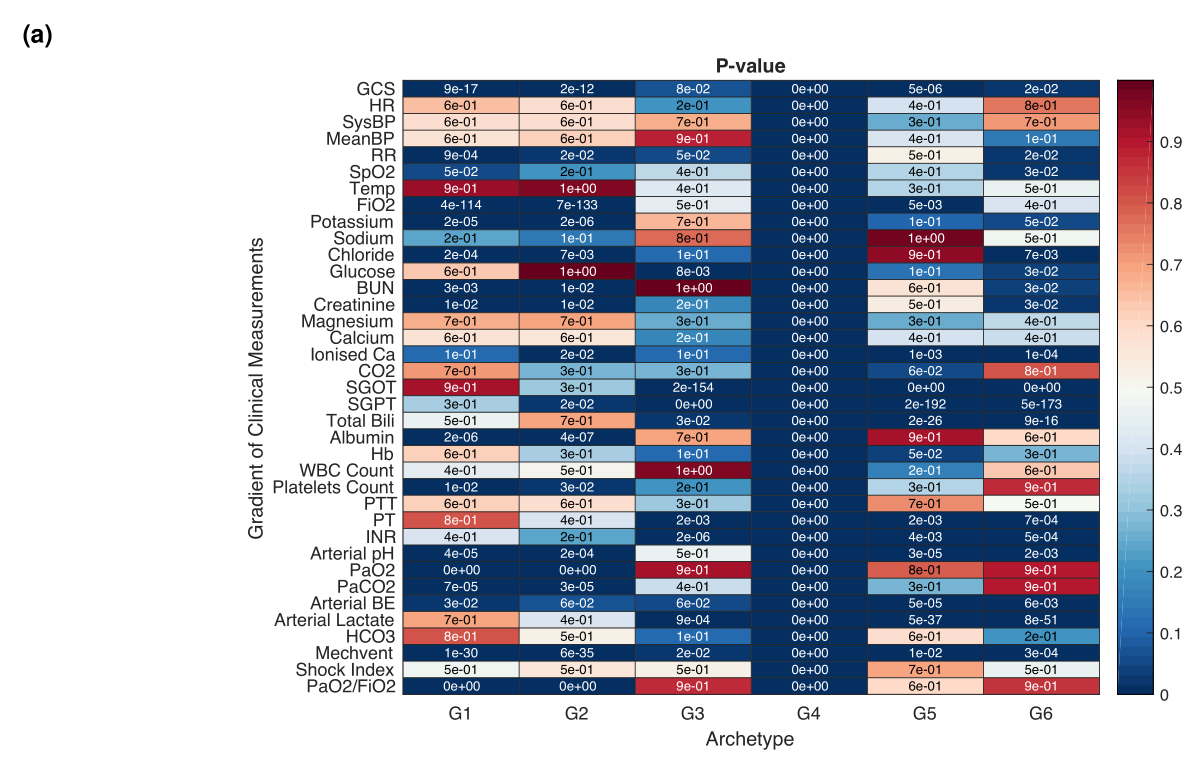

Supplement: S5 Fig — (A) P-values for the pairwise two-sample t-test for the gradients of clinical measurements (row) of each gradient group (column). Statistically significant entries approach blue. (TIFF) [file pdig.0000130.s006.tiff]

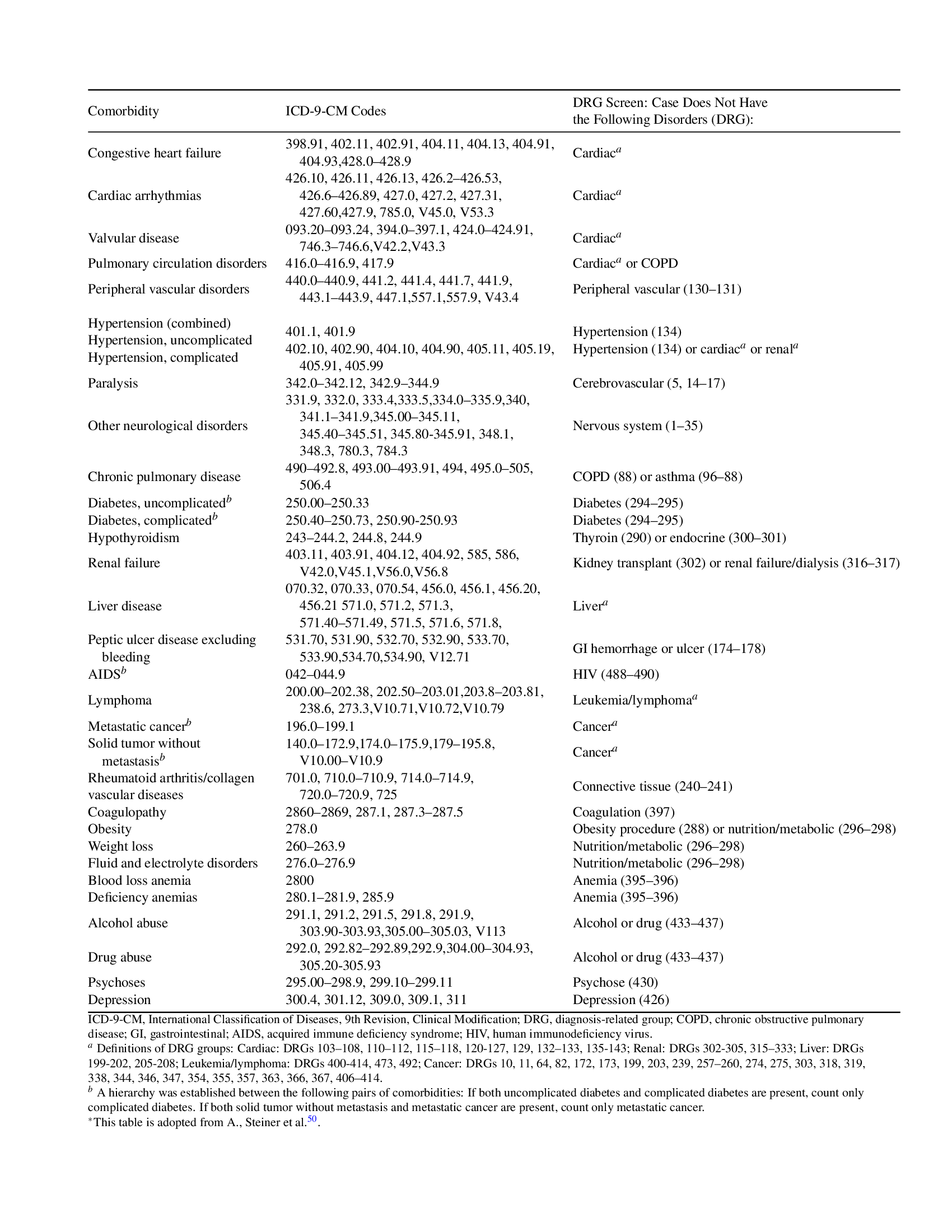

Supplement: S6 Table — (TIFF) [file pdig.0000130.s012.tiff]
